# Epidemiological investigation of feline chronic gingivostomatitis and its relationship with oral microbiota in Xi’an, China

**DOI:** 10.3389/fvets.2024.1418101

**Published:** 2024-06-14

**Authors:** Pengxiu Dai, Mingxia Yang, Juanjuan Du, Keyi Wang, Ruiqi Chen, Xiancheng Feng, Chen Chen, Xinke Zhang

**Affiliations:** ^1^College of Veterinary Medicine, Northwest A&F University, Yangling, Shaanxi, China; ^2^MOA Key Laboratory of Animal Virology, Center for Veterinary Sciences, Zhejiang University, Hangzhou, Zhejiang, China; ^3^Department of Veterinary Medicine, College of Animal Sciences, Zhejiang University, Hangzhou, Zhejiang, China

**Keywords:** feline chronic gingivostomatitis, epidemiological investigation, oral microbiota, *Porphyromonas*, drug susceptibility test

## Abstract

Feline chronic gingivostomatitis (FCGS) is an ulcerative and/or proliferative disease that typically affects the palatoglossal folds. Because of its unknown pathogenesis and long disease course, it is difficult to treat and has a high recurrence rate. Most of the bacteria in the oral microbiota exist in the mouth symbiotically and maintain a dynamic balance, and when the balance is disrupted, they may cause disease. Disturbance of the oral microbiota may play an important role in the development of FCGS. In this study, the medical records of 3109 cats in three general pet hospitals in Xi ‘an were collected. Sixty-one cats with FCGS were investigated via questionnaires, routine oral examinations and laboratory examinations. Oral microbiota samples were collected from 16 FCGS-affected cats, and microbial species were identified by 16S rDNA sequencing. The results showed that the incidence of FCGS had no significant correlation with age, sex or breed. However, the incidence of FCGS was associated with immunization, a history of homelessness and multicat rearing environments. The number of neutrophils and the serum amyloid A concentration were increased, and the percentage of cells positive for calicivirus antigen was high in all cases. All the cats had different degrees of dental calculus, and there were problems such as loss of alveolar bone or tooth resorption. Compared with those in healthy cats, the bacterial diversity and the abundance of anaerobic bacteria were significantly increased in cats with FCGS. *Porphyromonas*, *Treponemas* and *Fusobacterium* were abundant in the mouths of the affected cats and may be potential pathogens of FCGS. After tooth extraction, a shift could be seen in the composition of the oral microbiota in cats with FCGS. An isolated bacteria obtained from the mouths of the affected cats was homologous to *P. gulae*. Both the identified oral microbiota and the isolated strain of the cats with FCGS had high sensitivity to enrofloxacin and low sensitivity to metronidazole. This study provides support to current clinical criteria in diagnosing FCGS and proposes a more suitable antibiotic therapy.

## Introduction

Stomatitis is the general term for oral mucosal inflammation, a common oral disease in cats; its incidence is second only to that of periodontal disease and is described as a proliferative and ulcerative inflammation of the oral cavity (1). Feline chronic gingivostomatitis (FCGS) is a chronic disease typically affecting the palatoglossal folds (2). Oral pain, loss of appetite, halitosis, salivation, depression and weight loss are some of the clinical signs of FCGS and seriously affect the quality of life of cats. Because of its unknown pathogenesis and slow course, it is currently the most difficult to treat stomatitis. FCGS constitutes, a commonly diagnosed disease in cats, with a incidence of 0.7–12.0%, and can occur at all ages after tooth replacement (3). Feline chronic gingivostomatitis, an oral mucosal disease of unknown etiology that is difficult to cure, is distinct from gingivitis. The inflammation associated with FCGS extends beyond the gingival junction to the buccal and oral mucosa (4). With further research, it is generally believed that FCGS is caused by an inappropriate immune response caused by antigen stimulation, which may be caused by multiple factors in nature, such as bacterial infection, viral infection, other dental diseases, hypersensitivity reactions, and food allergies (5, 6).

The microbial diversity in the oral cavity is high. Bacteria not only attach to the oral surface but also form multigenus communities through mutual adhesion. Most of these resident microbiomes exist in the oral cavity symbiotically, not only causing no harm to the body but also controlling pathogenic bacteria to maintain the dynamic balance through bacterial membranes (7). When the dynamic balance in the mouth is disrupted, pathogenic bacteria or opportunistic pathogens overgrow and reproduce in the mouth, gradually affecting the teeth themselves and the soft tissues of the oral mucosa, which can cause certain common oral diseases, such as dental caries, gingivitis and periodontitis (8, 9). Some studies believe that bacteria play a certain role in the pathogenesis of FCGS. In relevant studies on the oral bacteria associated with FCGS, different experimental results have been reported. One study reported that the oral microbiota diversity of cats with FCGSs was greater than that of healthy cats (10). Some studies have also reported that the diversity of oral microbiota in cats with FCGSs did not significantly change or even decreased (10–14), but all reports had one thing in common; that is, the detection rate of anaerobic bacteria in the oral microbiota of cats with FCGSs was significantly greater than that of healthy controls. The key role of oral microbiota disturbance in the occurrence and development of FCGSs and the extent to which changes in the oral microenvironment influence the prognosis of FCGSs remain to be further studied.

In this study, the medical records of 3,109 cats with FCGSs were collected from three general pet hospitals in Xi’an. Sixty-one cats with FCGS were investigated via questionnaires, routine oral examinations and laboratory examinations. The incidence and characteristics of FCGS were analyzed to propose theoretical support for the prevention, control and diagnosis of this disease. Oral microbiota samples were collected from 16 cats with FCGS, and microbial species were identified by 16S rDNA sequencing. The differences in the oral microbiota species and abundance between healthy cats and cats with FCGS as well as between cats before and after tooth extraction treatment were evaluated, and possible characteristic pathogens were explored. At the same time, the sensitivity of cat oral microbiota to commonly used clinical antibiotics was evaluated, potential pathogenic bacteria were isolated and identified, and drug sensitivity tests were conducted *in vitro* to propose a drug reference for the treatment of FCGS.

## Methods

### Epidemiological investigation

This was a prospective study, from September 2021 to September 2022, 3,109 cats admitted to Xi’an Animal Hospital of Northwest Agriculture and Forestry University, Xi’an Hemeng Pet Animal Hospital and Xi’an Healing International Animal Hospital were collected, and cases clinically diagnosed with FCGS were taken as the research objects to conduct an epidemiological investigation of FCGS. Through medical history investigations and clinical examinations, patients who met the following characteristics of chronic gingivostomatitis stomatitis were included in this study: (1) long-term oral mucosal inflammation lasting several months to several years; (2) oral inflammation extending beyond the gingival mucosa to the alveolar mucosa and oropharyngeal side to the palatoglossal fold; (3) the severity of mucosal inflammation is more intense than that which can be caused by current dental disease; (4) edema, hyperplasia and/or ulceration at the site of inflammation; and (5) the inflamed area is bright red, and even spontaneous bleeding or very light touch (such as a swab) can cause bleeding (15, 16). At the same time, the cat oral examination record form should be completed (Additional file 1) (17, 18).

### Laboratory examination

Blood was collected from the great saphenous vein of the hind limb of 32 cats with FCGS and placed in an anticoagulation tube. Routine blood tests were performed using a fully automated five-category blood cell analyzer (IDEXX, ProCyte Dx, United States). An automatic nucleic acid detection system (GlinX, InCycle, China) was used to detect feline immunodeficiency virus and feline leukemia virus. Serum was obtained by centrifugation of the cat blood samples, and serum amyloid A (SAA) was examined by an immunofluorescence quantification analyzer (Boditech Med, VET CHROMA, Korea). A disposable sterilized cotton swab was used to collect secretions from the eyes, nose and mouth of each infected cat, which were added to 1 mL of sample storage solution. Then, the cotton swab stem was broken, and the cap was tightened and thoroughly mixed. An automatic nucleic acid detection system (GlinX, InCycle, China) was used for the nucleic acid detection of feline calicivirus and feline herpesvirus.

After the cats with FCGS were anesthetized with propofol, tracheal intubation was performed via a ventilator and anesthesia machine (Mindray, WATO-EX-20Vet, China), and isoflurane was used for inhalation anesthesia. After general anesthesia was stabilized, full-mouth dental X-rays were taken (Genoray, EZX-60, China). The presence of alveolar bone loss, resorption, fracture and root retention were evaluated.

In the tooth extraction treatment of cats with FCGS, on the premise of not affecting the operation of the surgeon or the treatment and prognosis of the cat, pathological mucosal tissue 5 mm (length) × 2 mm (width) × 2 mm (thickness) in size was clipped and placed in 4% paraformaldehyde for fixation. Tissues of the same size were collected from the healthy oral mucosa of domestic cats who died from other diseases and fixed in 4% paraformaldehyde as a control. After sampling, 5–0 single-strand PGA sutures were used to close the mucosal incision. The samples were sliced and stained with hematoxylin–eosin and then observed under a microscope (Nikon, Ni-U, China).

### 16S amplicon sequencing analysis

Among the 61 cats with chronic gingivitis who underwent epidemiological investigation, 16 cats with FCGS requiring half-mouth/full-mouth extraction were randomly selected as the disease group. Before tooth extraction, samples were taken from the oral lesion site, namely, the palatine tongue fold on both sides, with a sterile cotton swab. The swab was promptly placed in a sterile EP tube filled with 1 mL of sterilized saline. The samples were stored at −80°C and recorded as the PGB group. On the 45th day after tooth extraction, the second sample was taken and denoted as the PGA group. Six healthy adult cats were randomly selected as the control group. After confirming that the patient had no oral disease, samples were taken and recorded as the NG group. The samples were sent to Beijing Novogene Technology Co., Ltd., for 16S amplicon sequencing, the raw data were obtained (PRJNA1092748), and quality control of the raw data was performed. After quality control, the DADA2 module in QIIME2 software was used to reduce noise, and sequences with abundances less than 5 were filtered out to obtain amplicon sequencing variants (ASVs) (19). Species annotations were made for each ASV using a pretrained naive Bayes classifier and QIIME2’s classify-sklearn algorithm (20, 21). The alpha diversity was calculated using seven indicators (Observed_otus, Chao1, Shannon index, Simpson index, dominance, Good’s coverage, and Pielou’s index) (22). Beta diversity was calculated based on weighted and unweighted distances (23). Adonis and Anosim functions were used to analyze the differences in community structure between groups (24, 25). The significantly different species were analyzed by MetaStat, t tests and LEfSe software (26). Functional annotation analysis was performed using functional annotation of prokaryotic taxa (Tax4Fun) (27).

### Oral microbiota drug sensitivity test

The oral mucosal lesions (palatine tongue fold) of 16 cats were sampled with sterile cotton swabs, and the swabs were promptly placed in EP tubes containing 1 mL of sterilized saline. Samples from the EP tubes were transferred to LB liquid medium (Solarbio, China) (aerobic culture) and liquid paraffin-sealed BHI broth medium (Solarbio, China) (anaerobic culture) on a benchtop (Antai Air Technology, China). The cultured samples were uniformly coated on the surface of blood plate medium (Huankai, Microbial, China) for the drug sensitivity test. Amoxicillin, ceftriaxone, cefalexin, azithromycin, gentamicin, doxycycline, enrofloxacin and metronidazole drug sensitive test paper (Binhe Microbial Reagent, China) were selected for the drug sensitivity test. 24 h later, after the bacteriostatic ring formed on the surface of the medium, the diameter of the bacteriostatic ring was measured with Vernier calipers and recorded.

### Isolation and culture of *Porphyromonas*

First, the initial culture was carried out. The samples collected in a 1 mL normal saline EP tube were transferred to a BHI broth tube with a paraffin seal layer on a benchtop for culture for an additional 6 h. The bacterial liquid layer was gently removed with a pipette, 50 μL of bacterial suspension was added, and the mixture was diluted 10^−1^, 10^−2^, 10^−3^, 10^−4^, and 10^−5^ times. Fifty microliter suspensions with different dilutions were coated in BHI blood agar medium with a triangle rod and labeled. The plates were placed in an anaerobic tank and then cultured in an anaerobic production bag with an anaerobic indicator at 37°C for 5 ~ 7 days. Then, pure culture was carried out, colony morphology was observed, black round colonies with smooth surfaces were selected, and the bacteria were separated through a three-zone drawing line. After 4 to 5 generations, the colony characteristics were observed until the colony morphology became uniform.

### Identification of *Porphyromonas*

The morphological and culture characteristics, such as size, color, luster, texture, uplift state and edge characteristics, of the isolated and purified colonies were observed. Single colonies were selected for Gram staining and observed under a microscope. The target single colony was selected, and colony PCR was performed using universal primers for bacterial 16S rDNA (98°C, 10 min, 1 cycle; 98°C, 10 s, 55°C, 15 s, 72°C, 30 s, 35 cycle; 72°C, 10 min, 1 cycle). The PCR amplification products were subjected to agar gel electrophoresis, and the size of the product fragments was determined by a UV gel imaging system. The PCR-amplified products and primers were sent to Tsingke Biotechnology Co., Ltd., for sequencing. The sequencing results were compared and analyzed online via the BLAST program on NCBI to identify the isolated bacteria. The biochemical identification of the isolated strains was performed with an ATB Rapid ID 32A strip (BioMerieux, France) and an ATB Automatic Bacterial Identification instrument (BioMerieux, France).

The obtained strains and the 16S rRNA gene sequences of common reference strains in *Porphyromonas* were introduced into the MEGA X format in fasta format, and Clustal W was selected for DNA multi-sequence comparison. After comparison, the bases at the head and tail non-conserved sites were pruned to keep the sequences neat. Then the pruned sequence will be generated into meg format file and saved, the distance matrix will be calculated, the bootstrap value will be set to 1,000 to test the confidence, and the Poisson mode l model distribution will be selected for calculation. The phylogenetic tree based on 16S rRNA sequence gene was constructed.

### Determination of the minimum bacteriostatic concentration of drugs

Using a turbidimeter and a 0.5 McIntosh turbidity standard tube, the isolated strains were prepared in 0.5 McIntosh turbidity liquid. The gentamicin, amoxicillin, ceftriaxone, cefalexin, enrofloxacin, doxycycline and metronidazole preservation solutions were prepared at a concentration of 2560 μg/mL, divided into sterile EP tubes and stored at −20°C. The prepared drug preservation solution was diluted by the double dilution method, and the plate was prepared according to a ratio of drug to agar of 1:9. The plate was a BHI agar blood plate. The drug concentrations of the prepared BHI agar blood plates were 256, 128, 64, 32, 16, 8, 4, 2, 1, 0.25, 0.125, and 0.25 μg/mL, respectively, and the prepared media were stored at 4°C. A pipette was used to draw up 10 μL of 0.5 McIntosh turbidity isolated strain solution, which was inoculated on media supplemented with different concentrations of drugs. At the same time, a quality control strain solution (the standard strain *Clostridium perfringens* ATCC13124, provided by the Laboratory of Microbiology, College of Veterinary Medicine, Northwest A&F University) and a drug-free blank control were inoculated. After anaerobic culture at 37°C for 48 h, the lowest concentration observed for aseptic growth was the lowest antibacterial concentration, which was determined according to the CLSI standard.

## Results

### Epidemiological examination results

**Figure 1:**
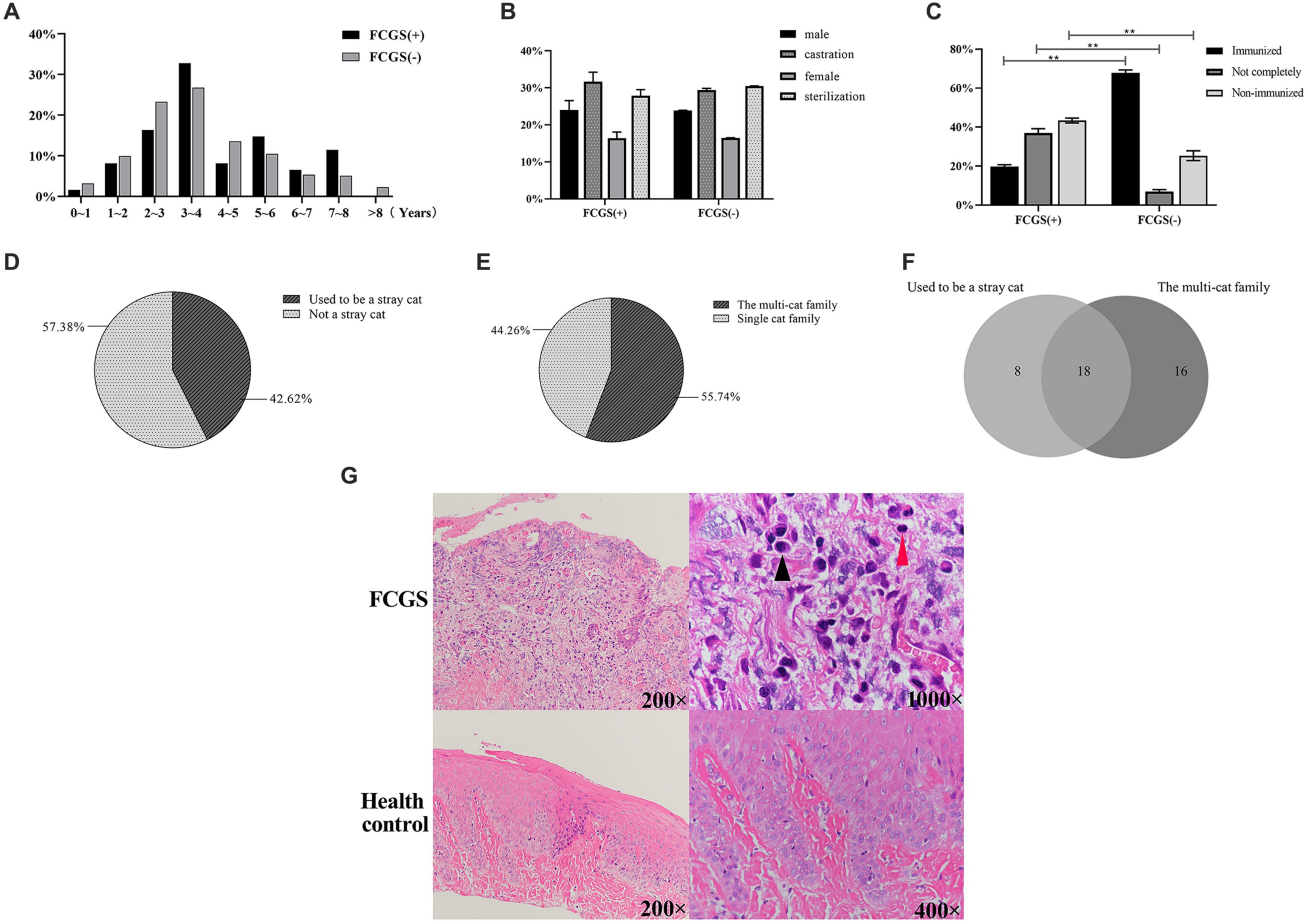
Epidemiological examination results. **(A)** Age distribution of cats in Xi’an. **(B)** Gender distribution and composition ratio of feline with FCGS in Xi’an. **(C)** Distribution and composition ratio of immunization of feline with FCGS in Xi’an. Statistically significance was indicated: ^**^p < 0.01. **(D)** Distribution of homelessness of feline with FCGS in Xi’an. **(E)** Distribution of family status of cats with FCGS in Xi’an. **(F)** Relationship between vagrancy situation and family status of cats with FCGS in Xi’an. **(G)** Results of hematoxylin–eosin staining of oral mucosa. The black arrows indicate plasma cells and the red arrows indicate lymphocytes.

The statistical results showed that among the 3,109 cats admitted to three animal hospitals in Xi’an, 61 were clinically diagnosed with FCGS, accounting for 1.96%, indicating a high incidence. Among them, 36 cats were revisited after receiving treatment, accounting for 59.02% of the total, indicating that FCGS has a high recurrence rate and is difficult to cure. The clinical symptoms of FCGS in the survey included difficulty in swallowing/chewing, halitosis, salivation, bleeding of the oral mucosa, unkempt fur, and weight loss (Table 1). The data of the 61 cats with FCGS and 2,969 cats without FCGS were collected at this time, and the ages of the samples were statistically analyzed at intervals of 1 year. There was no significant difference in the age distribution of cats with and without FCGS, and the specific distribution is summarized in Figure 1A. Statistical analysis of sex information collected from the 61 affected cats revealed that the ratio of females to males was 1:1.26. After excluding FCGS cases and cases with unknown sex, the ratio of females to males was 1:1.04 in a total of 2,987 non-FCGS cases, showing no significant correlation between FCGS and sex (Figure 1B).

**Table 1:** Distribution and proportion of clinical symptoms in cats with FCGS in Xi’an.

Clinical symptom	Quantity/example	Percentage
Difficulty swallowing/chewing	50	81.97%
Halitosis	49	73.77%
Salivation	31	50.82%
Weight loss	24	39.34%
Bleeding of the oral mucosa	14	31.15%
Unkempt fur	17	27.87%

According to the statistical analysis of the data collected from the 61 cats with FCGS, the proportions of cats affected according to the different breeds were different. The proportion of Chinese garden cats was the highest, at 59.02% (36/61), followed by Siamese cats at 13.11% (8/61) and Ragpet cats at 13.11% (8/61), among which the proportion of Gabby cats was the lowest (Table 2).

**Table 2:** Distribution and composition ratio of FCGS affected cat breeds in Xi’an.

Breed	Quantity/example	Composition ratio
Chinese garden cats	36	59.02%
Siamese cats	8	13.11%
Ragpet cats	8	13.11%
British shorthair	5	8.20%
American shorthair	3	4.92%
Exotic shorthair	1	1.64%

According to the vaccination status of the infected cat as known by the animal owners, the cases were classified as immunized, not fully immunized, or not immunized. Cats that had been vaccinated according to the vaccine protocols recommended by authoritative organizations such as WSAVA (World Association of Small Animal Veterinarians) and AAHA (American Animal Hospital Association) were classified as immunized. Cats that were not fully vaccinated according to the vaccine protocol were classified as incompletely immunized; cats that had never been vaccinated and stray cats with unknown medical histories that had not been vaccinated after rescue were classified as non-immunized. According to the statistics of the 61 infected cats investigated, the proportion of unimmunized cats was the highest at approximately 42.62% (26/61), followed by the proportion of incompletely immunized cats at approximately 37.70% (23/61). Differential expression analysis revealed significant differences between non-immunized, incompletely immunized, and immunized cats with and without FCGS (*p* < 0.01) (Figure 1C).

The animal owners were asked if their cat had a history of homelessness, that is, whether it was a stray cat that had been adopted or rescued or had lived alone outside the home. Among the 61 cats with FCGS from which samples were collected, 26 had a history of homelessness, accounting for approximately 42.62%, and 35 cases had no history of homelessness, accounting for 57.38% (Figure 1D). Households with two or more cats were classified as multicat households; if there was only one cat in the household, it was classified as a single-cat household. Among the 61 cats with FCGSs, 55.74% (34/61) were from multicat families. The number of patients from single-cat families was relatively small, accounting for approximately 44.26% (27/61) (Figure 1E). According to the above statistics, cats with FCGS who had a history of homelessness and who belonged to multicat families accounted for a greater proportion of the sample. According to the Venn diagram of the two influencing factors, there were 18 cats with FCGS with a history of homelessness and belonging to multicat families, accounting for 29.51% of the total number of cases. Cats with a history of homelessness are often adopted by their owners as domestic cats, and there are often two or more cats in the household, resulting in a large overlap between the two factors (Figure 1F). Of the 61 cases of FCGS in this epidemiological investigation, 52 cats had varying degrees of dental calculus (Table 3).

**Table 3:** Grade distribution and composition ratio of dental calculus of cats with FCGS in Xi’an.

Dental calculus grade	Quantity/example	Composition ratio
0	0	0%
1	4	7.69%
2	18	34.62%
3	30	57.69%

Blood samples were collected from 32 cats with FCGS. The routine blood indicators of the cats were within the normal ranges, and the total white blood cell count was significantly. In the white blood cell system, more specifically, only the number of neutrophils was significantly increased, and other indicators were within the normal ranges (Table 4). Among the 32 cats with FCGS from which samples were collected, the mean SAA was 51.73, showing a significant increasing trend (Table 5). Virus detection was performed on 18 cats with FCGS, 16 of which were feline calicivirus (FCV) antigen positive, 3 of which were feline herpesvirus (FHV) antigen positive, and 1 of which was feline immunodeficiency virus (FIV) antigen positive. No feline leukemia virus (FeLV) antigen positivity was detected, and one of the cats was co-infected with FCV and FHV.

**Table 4:** In the complete blood count of cats affected by FCGS.

Items	FCGS *X* ± *SD*	Reference range
RBC (10^12^/L)	8.72 ± 1.59	6.54–12.20
HCT (%)	38.87 ± 7.70	30.3–52.3
HGB (g/dL)	13.62 ± 2.34	9.8–16.2
MCV (fL)	44.77 ± 5.52	35.9–53.1
MCH (Pg)	14.84 ± 1.67	11.8–17.3
MCH (g/L)	33.37 ± 1.44	28.1–35.8
RDW (%)	18.42 ± 4.15	15.0–27.0
RETIC (K/μL)	21.61 ± 8.80	3.0–50.0
WBC (10^9^/L)	18.21 ± 9.58↑	2.87–17.02
NEU (10^9^/L)	12.78 ± 8.23↑	2.30–10.29
LYM (10^9^/L)	4.28 ± 1.95	0.92–6.88
MONO (10^9^/L)	0.49 ± 0.31	0.05–0.67
EOS (10^9^/L)	0.63 ± 0.38	0.17–1.57
BASO (10^9^/L)	0.02 ± 0.03	0.01–0.26
PLT (K/μL)	238.90 ± 82.89	151–600
MPV (fL)	14.64 ± 2.74	11.4–21.6
PCT (%)	0.30 ± 0.17	0.17–0.86

↑Indicates that the value exceeds the reference value.

**Table 5:** SAA concentration in cats with FCGS in Xi’an.

SAA grading	Quantity/example	Composition ratio
<10 μg/mL	10	31.25%
10–50 μg/mL	9	28.13%
50–200 μg/mL	11	34.38%
≥200 μg/mL	2	6.25%

In the 32 dental X-rays collected from cats with FCGS, multiple dental problems were present at the same time (Table 6). Persistent and widespread inflammation in the mouths of cats with FCGS may be accompanied by extensive alveolar bone loss, tooth resorption, and tooth root retention. Full dental radiography is therefore a critical component of the diagnosis of a cat undergoing treatment.

**Table 6:** Distribution and proportion of dental problems in cats with FCGS in Xi’an.

Type	Quantity/example	Percentage
No obvious abnormality	3	9.38%
Horizontal loss of alveolar bone	27	84.38%
Vertical loss of alveolar bone	6	18.75%
Tooth resorption	25	78.13%
Tooth fracture	1	3.13%
Retained dental root	2	6.25%

Oral mucosal tissue sections showed no obvious epithelial abnormalities in healthy cats and no inflammatory cell infiltration in the lamina propria. The epithelial morphology of cats with FCGS was disorganized and incomplete, and the upper tissue layer was separated from the lamina propria. Obvious inflammatory cell infiltration, mainly plasma cells and lymphocytes, was observed in the lamina propria, accompanied by massive neovascularization (Figure 1G).

### ASV clustering results

**Figure 2:**
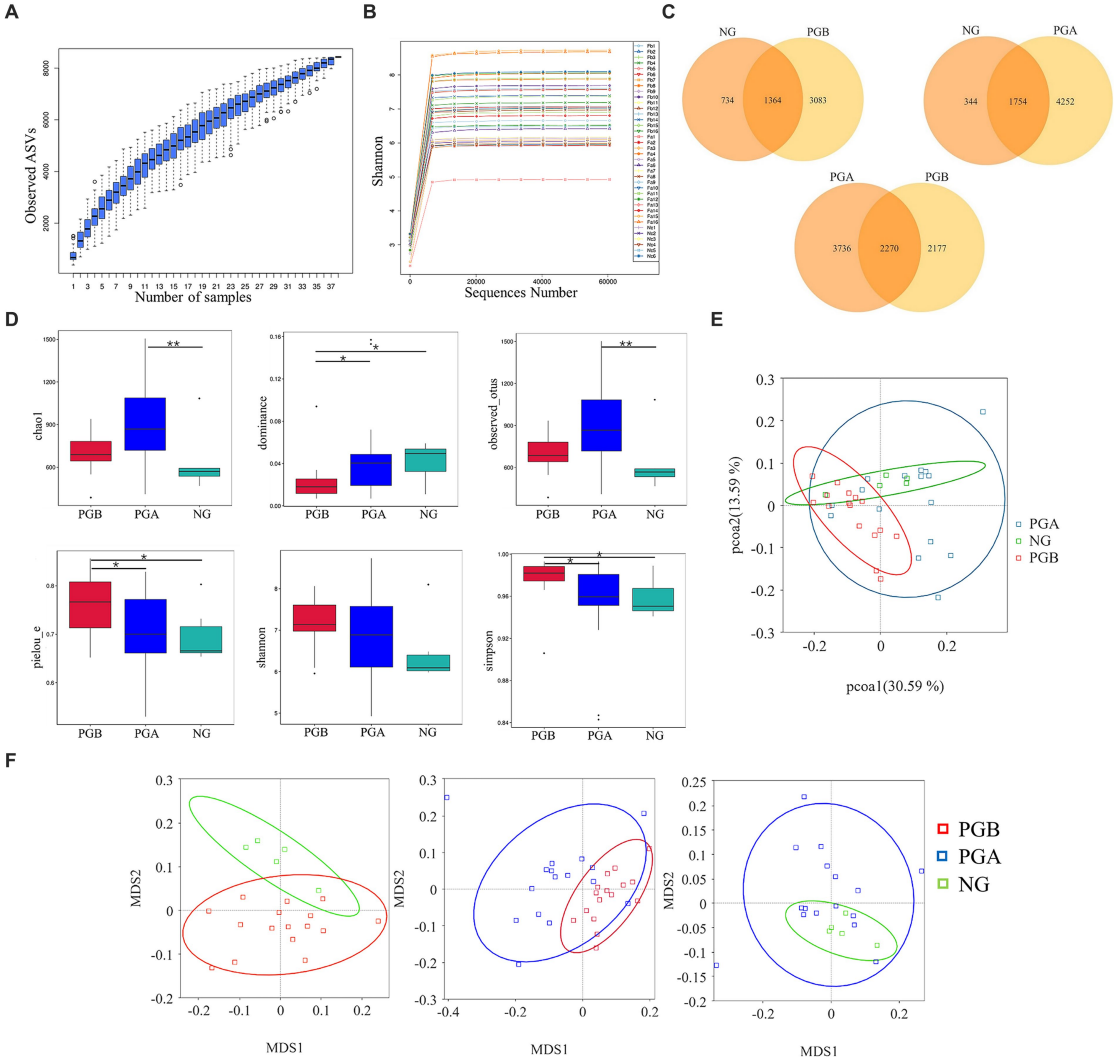
Diversity analysis results. **(A)** Species accumulation boxplot. **(B)** Rarefaction Curve (b) of oral microbiota samples. **(C)** Venn Graph of oral microbiota ASVs. **(D)** Alpha Diversity Index Box Plot of difference between groups. Statistically significance was indicated: *P<0.05; **P<0.01. **(E)** Weighted unifrac PCoA Two-dimensional diagram. **(F)** Weighted unifrac distance NMDS chart.

A total of 38 oral samples were collected from 16 cats with FCGS (pre- and post-operative) and 6 healthy cats. As the number of samples increased, the species accumulation curve became relatively flat, indicating that the probability of different ASVs occurring decreased with increasing sample size, and the sample size met the analysis requirements of this study (Figure 2A). The dilution curve directly reflected the rationality of the amount of sequencing data and indirectly reflected the species richness of the samples. As the curve tended to be smooth, the amount of sequencing data was reasonable (Figure 2B). To avoid sequencing errors or random factors affecting the analysis results, ASVs with low abundance were filtered, and the remaining ASVs were used for subsequent analysis.

A total of 8,433 ASVs were detected in all the samples after noise reduction. A total of 4,447, 6,006 and 2098 ASVs were obtained from the PGB, PGA and NG samples, respectively. Overall, 3,083 and 734 unique ASVs were detected in the PGB and NG groups, respectively. A total of 2,177 and 3,736 unique ASVs were found in the PGB and PGA groups, respectively. A total of 4,252 and 344 unique ASVs were found in the PGA and NG groups, respectively. As shown in the Venn diagram of the ASVs of the oral microbiota of infected cats and healthy cats, the overall number of ASVs of the oral microbiota of cats with FCGS was greater than that of healthy cats (Figure 2C). The more ASVs there were, the greater the species diversity there was. Therefore, the oral microbiota of cats with FCGS was more complex and diverse than that of healthy cats.

### Alpha diversity analysis results

In the intergroup difference analysis of the alpha diversity index, a box chart can directly reflect the intragroup species diversity, and the significance of intergroup species diversity differences can be analyzed through the Tukey and Kruskal–Wallis rank sum tests. According to the Chao1 index, the microbial richness of the PGA group was the highest, followed by that of the PGB group. The richness of oral samples in the NG group was relatively low, and the Chao1 index in the PGA group was significantly greater than that in the NG group (*p* < 0.01). Moreover, the microbial diversity of oral samples in the PGB group was greater than those in the other two groups, and there were significant differences in the dominance and Pielou_e indices between the PGB group and the PGA and NG groups (*p* < 0.05). The average Shannon index of the PGB group was approximately 7.1897, while those of the PGA and NG groups were approximately 6.9147 and 6.4613, respectively, with no significant difference among all groups (*p* > 0.05). The Simpson indices of the PGB, PGA and NG groups were 0.9768, 0.9513 and 0.9582, respectively, and the Simpson index of the PGB group was significantly greater than those of the PGA and NG groups (*p* < 0.05) (Figure 2D). The microbial diversity in the PGB group was greater than those in the PGA and NG groups, indicating that the composition of the oral microbiota in cats in the FCGS group changed and that the diversity increased.

### Beta diversity analysis results

Differences between different groups were detected via beta diversity index difference analysis, principal coordinate analysis and non-metric multidimensional calibration methods. Based on the weighted UniFrac distance, PCoA was performed for the three groups. The oral microbial community of cats with FCGS in the PGB group was clustered together, and the oral microbial community of healthy cats in the NG group was also relatively clustered, while the oral microbial community of PGA cats after tooth extraction was not obvious or dispersed (Figure 2E). This indicated that there were significant differences in the oral microbiota composition among the three groups. The intergroup and intragroup differences between samples were evaluated by NMDS analysis, and the stress was less than 0.2, indicating that NMDS could accurately reflect the degree of differences between samples. Pair-to-pair analysis and comparisons were carried out on the PGB, PGA, and NG groups. The clusters of the PGB and NG groups were significantly different. The overlap areas between the PGB and PGA groups and between the PGA and NG groups were large, and the bacterial community structures at these sites were similar (Figure 2F). There were significant differences in the composition of the oral microbiota among the three groups. According to the ANOSIM analysis, *r* > 0 and *p* < 0.05 were found between the oral microbiota of the PGB and PGA groups, indicating that there were significant differences in the oral microbiota between cats with FCGS and cats with FCGS after tooth extraction treatment, and the grouping was significant. The oral microbiota of the PGB and NG groups had *r* > 0 and *p* < 0.05, indicating that the oral microbiota of the cats with FCGS were significantly different from those of the healthy cats, and the grouping was significant.

### Results of the relative abundance analysis

**Figure 3:**
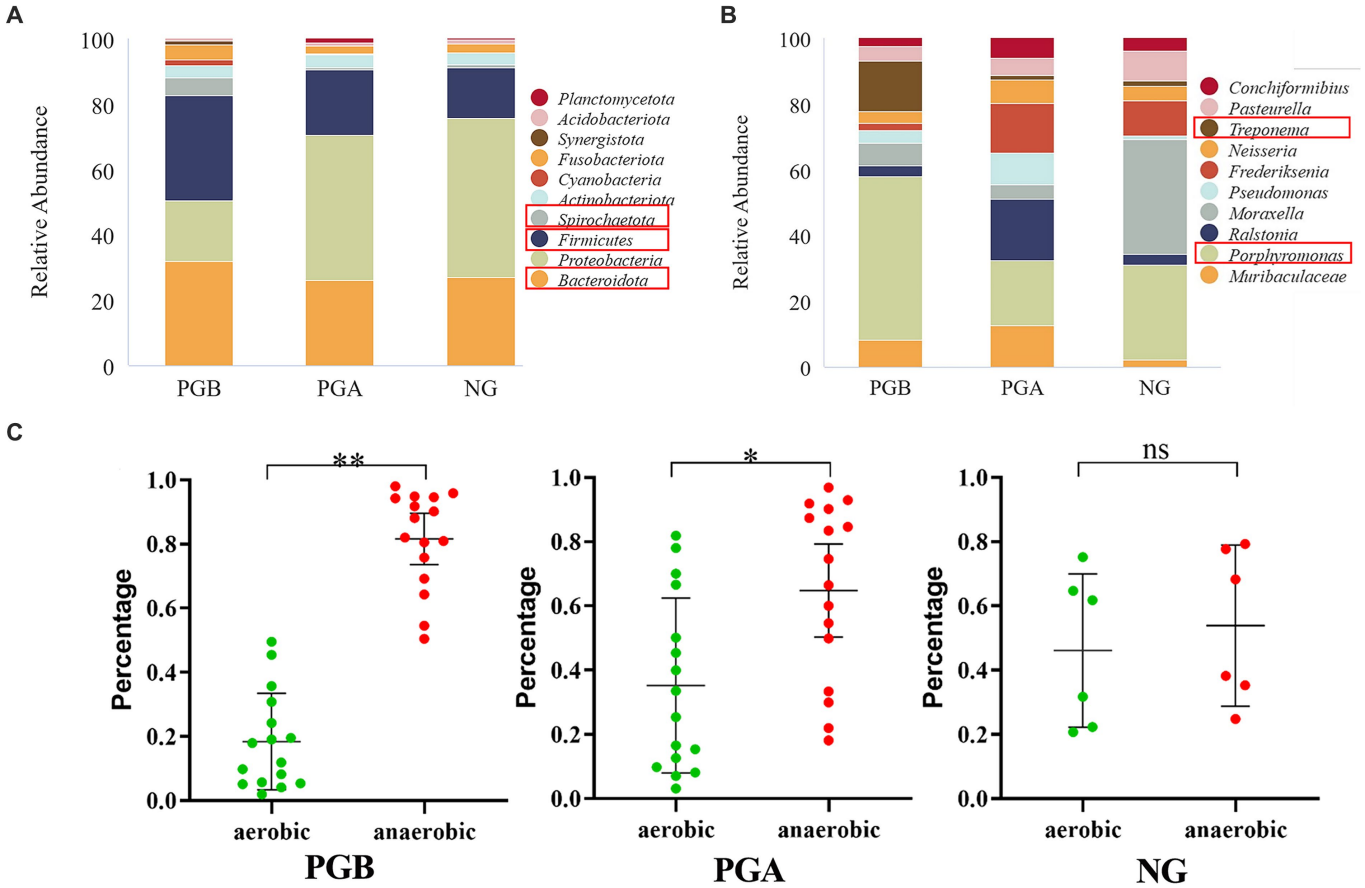
Results of relative abundance analysis. **(A)** The histograms of the highest abundance at the oral microbial phylum level (Top 10). **(B)** The histograms of the highest abundance at the oral microbial genus level (Top 10). **(C)** Differences in percentage abundance of anaerobic and aerobic bacteria. Statistically significance was indicated: ^*^p < 0.05, ^**^p < 0.01, ^ns^*p* > 0.05.

According to the above analysis of alpha diversity and beta diversity, there were significant differences in the composition of the oral microbiota among the three groups, in the abundance and diversity of the microbial communities, and in the heterogeneity of the species composition. At the phylum level, 43 phyla were clustered in the PGB group, among which *Firmicutes* was the most abundant. This was followed by *Bacteroidota*, *Proteobacteria*, *Spirochaetota*, *Firmicutes* and *Fusobacteria*. There were 43 phyla clustered in the PGA group. *Proteobacteria* had the highest bacterial abundance, followed by *Bacteroidota*, *Firmicutes*, *Actinobacteria* and *Fusobacteriota*. In the NG group, 32 phyla were clustered at the phylum level, and *Proteobacteria* had the highest abundance, followed by *Bacteroidota*, *Firmicutes*, *Actinobacteria* and *Fusobacteriota*, which was the same as that in the PGA group (Figure 3A). The relative abundance of cats with FCGS at the gate level (*Bacteroidota*, *Firmicutes*, and *Spirochaetota*) was greater than that of healthy cats; that is, these three gates may be potential pathogens.

The PGB group could be clustered into 737 genera at the genus level, and the most abundant genera were *Porphyromonas*, *Treponema*, *Muribaculaceae*, *Moraxella* and *Pasteurella*. There were 872 genera in the PGA group, among which *Porphyromonas*, *Ralstonia*, *Frederiksenia,* and *Muribaculaceae* had the highest bacterial abundance. In the NG group, 508 genera clustered at the genus level, of which *Moraxella* was the most abundant, followed by *Porphyromonas* and *Frederiksenia* (Figure 3B). The abundance of *Porphyromonas* in the PGB group was greater than those in the PGA and NG groups. The abundance of *Treponema* was greater in the PGB group but lower in the PGA and NG groups. In contrast, *Pasteurella* and *Moraxella* were more abundant in the NG group but less abundant in the PGB and PGA groups. *Porphyromonas* and *Treponema* may be potential pathogens of FCGS, while *Pasteurella* and *Moraxella* are common oral microbiota in healthy cats.

In the oral microbiota of cats in the PGB group, the abundance of anaerobic bacteria was significantly greater than that of aerobic bacteria (*p* < 0.001), and in the oral microbiota of cats in the PGA group after treatment, the abundance of anaerobic bacteria was significantly greater than that of aerobic bacteria (*p* < 0.05); however, there was no significant difference between the abundance of anaerobic and aerobic bacteria in the oral microbiota of healthy cats in the NG group (*p* > 0.05) (Figure 3C). The results showed that the proportions of aerobic and anaerobic bacteria in the mouths of cats with FCGS were significantly different from those in the mouths of healthy cats, and the proportion of anaerobic bacteria in the mouths of cats with FCGS increased. One month after tooth extraction, the abundance of anaerobic bacteria in the mouths of cats with FCGS decreased but was still greater than that in the mouths of healthy cats.

### Oral microbiota difference analysis

**Figure 4:**
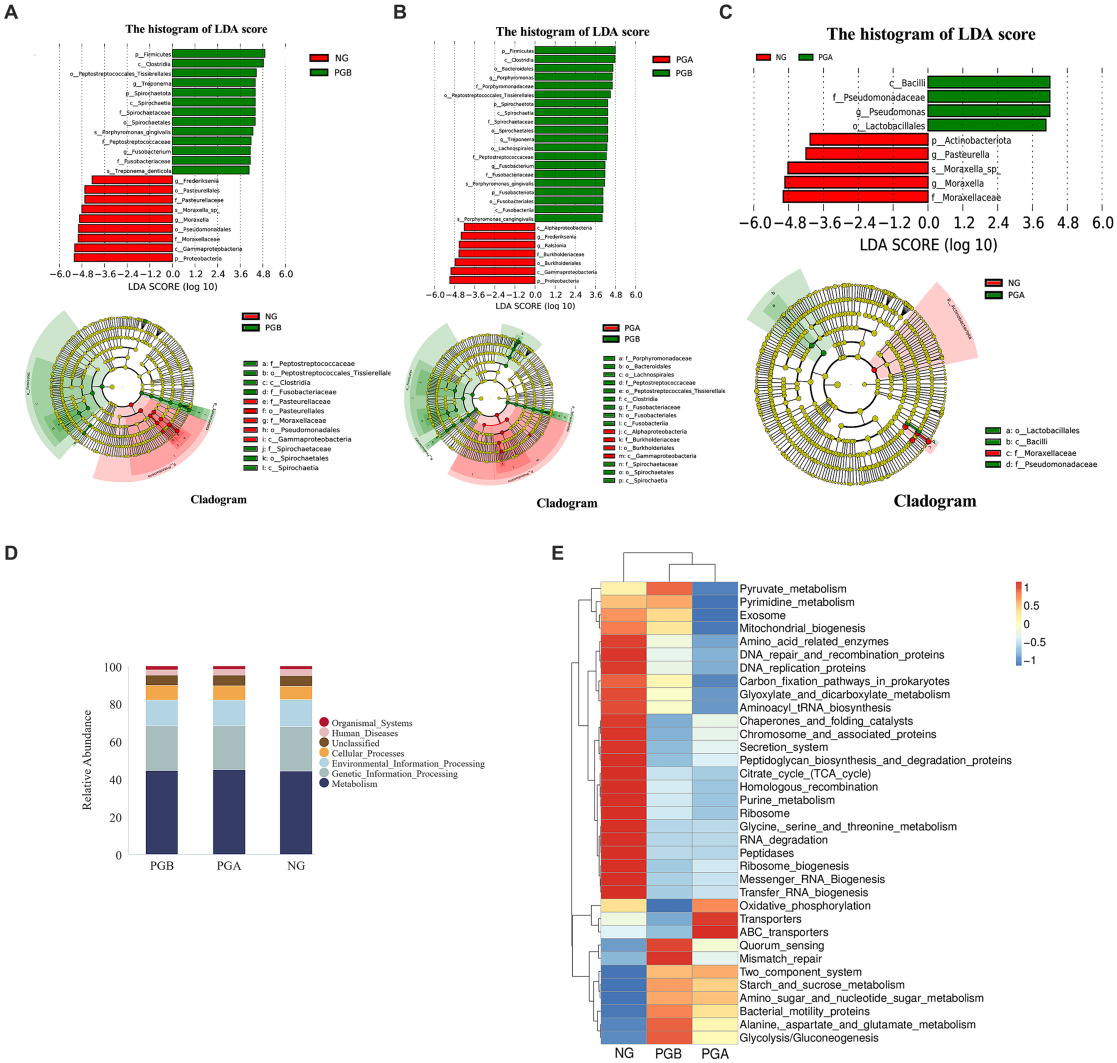
Species difference analysis and Tax4Fun function prediction results. **(A)** LEfSe results of oral microbiota in the PGB and NG groups. **(B)** LEfSe results of oral microbiota in the PGB and PGA groups. **(C)** LEfSe results of oral microbiota in the PGA and NG groups. **(D)** Histogram of predicted KEGG level 1 function of oral bacteria (Top 10). **(E)** Predicted heat map of KEGG level 3 function of oral bacteria in each group.

LEfSe analysis revealed different species between groups at different taxonomic levels. The relative abundances of *Firmicutes* and *Spirochaetota* in the PGB group were significantly greater than those in the NG group. The relative abundance of *Proteobacteria* in the NG group was significantly greater than that in the PGB group. Moreover, the relative abundances of *Treponema* and *Fusobacterium* in the PGB group were significantly greater than those in the NG group. The relative abundances of *Frederiksenia* and *Moraxella* in the NG group were significantly greater than those in the PGB group (Figure 4A). *Treponema* and *Fusobacterium* may be potential pathogens of FCGS.

The relative abundances of *Firmicutes*, *Spirochaetota* and *Fusobacteria* in the PGB group were significantly greater than those in the PGA group. The relative abundance of *Proteobacteria* in the PGA group was significantly greater than that in the PGB group. Moreover, the relative abundances of *Porphyromonas*, *Treponema* and *Fusobacterium* in the PGB group were significantly greater than those in the PGA group. The relative abundances of *Frederiksenia* and *Ralstonia* in the PGA group were significantly greater than those in the PGB group (Figure 4B). *Porphyromonas, Treponema,* and *Fusobacterium* are potential pathogens of FCGS.

The relative abundance of *Actinomycetes* in the NG group was significantly greater than that in the PGA group. Moreover, the relative abundance of *Pseudomonas* in the PGA group was significantly greater than that in the NG group. The relative abundances of *Pasteurella* and *Moraxella* in the NG group were significantly greater than those in the PGA group (Figure 4C).

### Tax4Fun function prediction results

Based on the Kyoto Encyclopedia of Genes and Genomes (KEGG) database 16S rRNA gene sequencing of whole prokaryote genomes, the sample function annotation and functional difference analysis of Tax4Fun were performed. The functional prediction results of the oral microbiota in the first KEGG level in the PGB, PGA, and NG groups are shown in Figure 4D. The major functional genes were related to metabolism, genetic information processing, environmental information processing, cellular process processes and organismal systems. There were no significant differences in the abundance of functional genes among all groups.

The functional prediction of the top 10 relative abundances of the oral microbiota in each group at the second KEGG level and their abundances are shown in Table 7. Carbohydrate metabolism and signal transduction gene abundances in the PGB group were greater than those in the NG group. The signal transduction gene expression in the PGA group was greater than that in the NG group, and carbohydrate metabolism was slightly greater than that in the NG group. The abundance of translation genes in the NG group was greater than those in the PGB and PGA groups.

**Table 7:** Functional prediction of KEGG level 2.

Items	PGB	PGA	NG
Carbohydrate metabolism	10.43%	10.24%	9.69%
Membrane transport	10.07%	10.36%	10.32%
Translation	9.78%	9.75%	10.26%
Replication and repair	9.55%	9.47%	9.77%
Amino acid metabolism	8.88%	8.83%	8.90%
Energy metabolism	4.48%	4.48%	4.46%
Nucleotide metabolism	4.18%	4.14%	4.22%
Metabolism of cofactors and vitamins	3.60%	3.57%	3.84%
Glycan biosynthesis and metabolism	3.31%	3.42%	3.65%
Signal transduction	2.91%	2.95%	2.46%

In the third-level function prediction heatmap (Figure 4E), the PGB and PGA groups were in the same subbranch, indicating that the two groups of function predictions had similar distribution rules. Multiple DEGs were enriched in the PGB group. The functional genes indirectly related to the immune system included genes related to the two-component signaling system and Mismatch_repair. There were two pathways involved in amino acid metabolism: alanine aspartate and glutamate metabolism and amino sugar and nucleotide sugar metabolism. The functional genes related to the regulatory mechanism of bacterial population behavior included Quorum-sensing and Bacterial motility proteins. There were two pathways involved in glucose metabolism: glycolysis/gluconeogenesis and starch and sucrose metabolism. The functional genes enriched in the PGA group included ABC transporters, oxidative phosphorylation and transporters.

### Drug sensitivity test results

The collected FCGS oral microbiota samples were cultured for drug sensitivity tests under aerobic and anaerobic conditions. The results of the drug sensitivity tests under aerobic conditions are shown in Table 8. The oral aerobic microbiota of cats with FCGS were highly sensitive to enrofloxacin; sensitive to doxycycline, gentamicin, ceftriaxone, cefalexin and amoxicillin; and insensitive to metronidazole. The results of the drug sensitivity tests under anaerobic conditions are shown in Table 9. The oral anaerobes of cats with FCGS were highly sensitive to enrofloxacin, doxycycline, amoxicillin and ceftriaxone and were generally sensitive to cephalexin, azithromycin and gentamicin, while the sensitivity to metronidazole was low. Overall, the oral microbiota of cats with FGCS were more sensitive to enrofloxacin and less sensitive to metronidazole.

**Table 8:** Results of the drug susceptibility testing for aerobic bacteria.

Classification	Drug sensitivity test strip	Diameter of the bacterial inhibition zone/mm diameter *X* ± *SD*	Sensitivity
Beta-lactam	Amoxicillin	15.56 ± 2.55	Highly sensitive
	Ceftriaxone	16.63 ± 4.53	Highly sensitive
	Cephalexin	12.50 ± 4.72	Moderate sensitivity
Macrolides	Azithromycin	14.81 ± 3.91	Moderate sensitivity
Aminoglycosides	Gentamicin	15.81 ± 5.85	Highly sensitive
Tetracycline	Doxycycline	15.94 ± 5.39	Highly sensitive
The quinolones	Enrofloxacin	20.06 ± 4.04	Super sensitive
Nitroimidazole	Metronidazole	3.06 ± 3.09	Low sensitivity

**Table 9:** Results of the drug susceptibility testing for anaerobic bacteria.

Classification	Drug sensitivity test strip	Diameter of the bacterial inhibition zone/mm diameter X ± SD	Sensitivity
Beta-lactam	Amoxicillin	18.19 ± 4.22	Highly sensitive
	Ceftriaxone	17.88 ± 4.90	Highly sensitive
	Cephalexin	13.94 ± 3.88	Moderate sensitivity
Macrolides	Azithromycin	12.94 ± 2.49	Moderate sensitivity
Aminoglycosides	Gentamicin	12.56 ± 2.45	Moderate sensitivity
Tetracycline	Doxycycline	17.63 ± 4.55	Highly sensitive
The quinolones	Enrofloxacin	19.13 ± 3.18	Highly sensitive
Nitroimidazole	Metronidazole	8.13 ± 1.49	Low sensitivity

### Isolation, identification and drug sensitivity test of *Porphyromonas*

**Figure 5:**
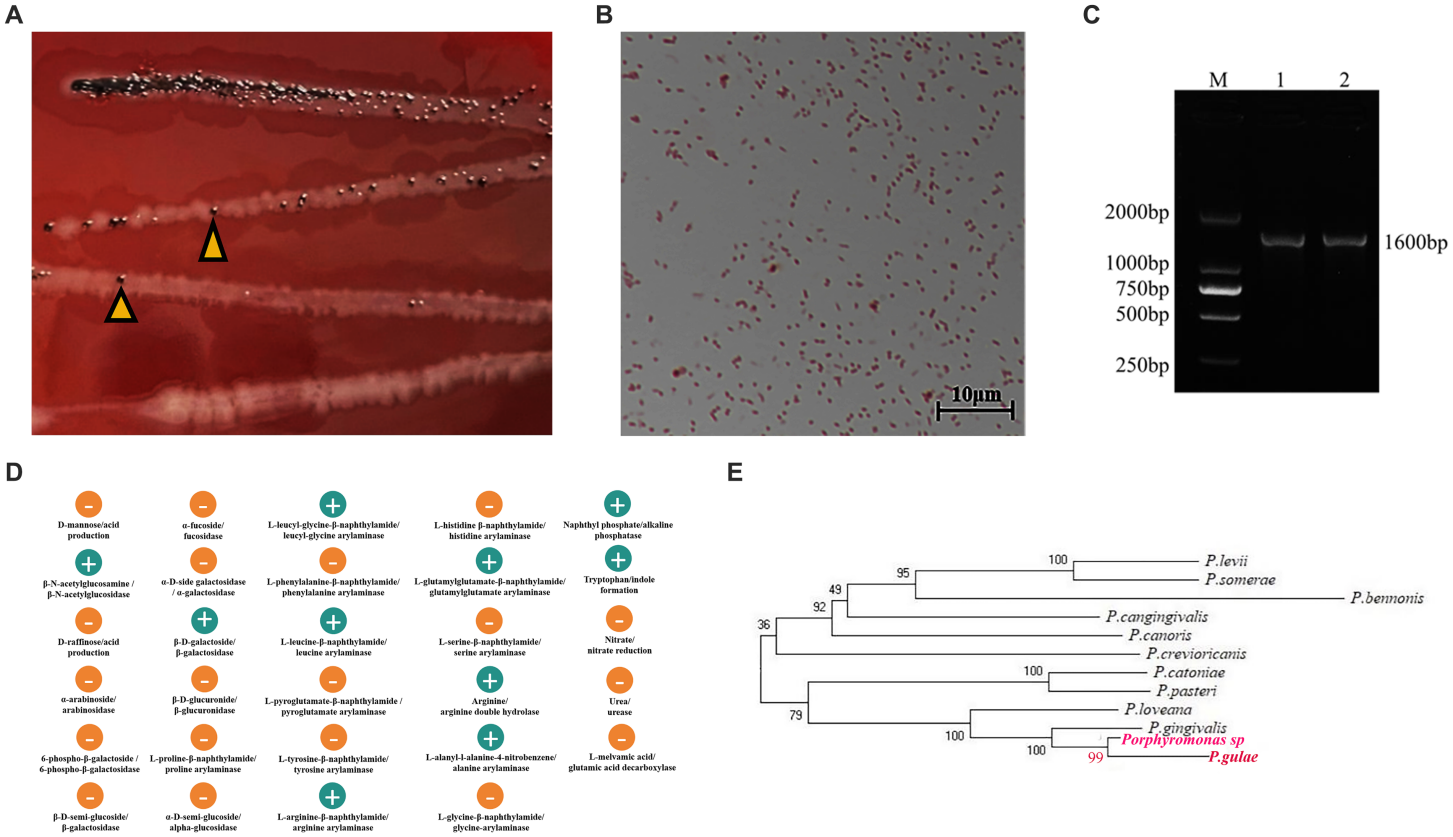
Isolation, identification and drug sensitivity test of *Porphyromonas*. **(A,B)** Colony morphology and microscopic observation results. **(A)** Colony morphology in BHI blood agar medium, the arrow refers to *Porphyromonas*. **(B)** Gram stain results. **(C)** PCR amplification results of isolated strains. M: DL2000 Marker; 1 and 2 are gel electrophoresis images of PCR products with universal primers. **(D)** The biochemical and metabolic reactions were analyzed by ATB Rapid ID 32A strip, and 29 biochemical reaction results were obtained. **(E)** According to evolutionary tree analysis, *Porphyromonas* sp. is 99% homologous to *P. gulae*, and both are 100% homologous to *P. gingivalis*.

One strain was isolated from the mouths of cats with FCGS, which was consistent with the morphology and staining characteristics of *Porphyromonas* colonies. The surface of the colony was smooth and moist, with a round bulge and black pigment, and the color changed from gray to black with a metallic luster around the 7th day of obligatory anaerobic culture (Figure 5A). After Gram staining, the strain appeared red (Figure 5B). The isolated strain was amplified by PCR, and 16S rDNA universal primers were used to generate 1,600 bp target bands (Figure 5C). After sequencing, online comparison and analysis by the BLAST program showed that the homology of the PCR product sequence and the sequence of the *Porphyromonas gulae* strain was 99%. The biochemical and metabolic reactions were analyzed by the ATB Rapid ID 32A strip, and 29 biochemical reaction results were obtained. The tested strain exhibited *β*-*N*-acetylglucosidase, *β*-galactosidase, arginine arylamidase, leucyl-glycine arylamidase, leucine arylamidase, alanine arylamidase, glutamylglutamate arylamidase, arginine bihydrolase, indole formation and alkaline phosphatase activity (Figure 5D). The results of the ATB Rapid ID 32A anaerobic bacteria biochemical test strip showed that the probability of the presence of the tested strain was 99.9% (%ID), and the *T*-value was 0.94. The resulting isolated strain was named *Porphyromonas* sp. According to evolutionary tree analysis, *Porphyromonas* sp. is 99% homologous to *P. gulae*, and both are 100% homologous to *P. gingivalis* (Figure 5E).

After the quality of the drug-containing agar blood plate was tested with a quality control strain, the MIC of the obtained strain was determined. The MICs of the five classes and seven drugs are shown in Table 10. According to the reference criteria, the isolate was sensitive to enrofloxacin, moderately sensitive to gentamicin, amoxicillin and ceftriaxone sodium, and resistant to cephalexin, doxycycline hydrochloride and metronidazole.

**Table 10:** MIC determination results for the *Porphyromonas* strain.

Classification	Drug name	MIC (μg/mL)	Result
Fluoroquinolones	Enrofloxacin	0.5–1	S
	Gentamicin	4–8	I
Aminoglycosides	Amoxicillin	4–8	I
Beta-lactam	Ceftriaxone sodium	16–32	I
	Cephalexin	>256	R
	Doxycycline hydrochloride	32–64	R
Tetracycline	Metronidazole	128–256	R

R (resistant), drug resistance; I (intermediate), moderately sensitive; S (sensitive), sensitive.

## Discussion

### Epidemiological investigation and analysis

In this study, 3,019 cats from 3 comprehensive animal hospitals in Xi’an were collected, among which 61 had FCGS, accounting for approximately 1.96%, indicating a high incidence. Due to the regional climate, dietary habits, oral microbiota and the time of case collection, the incidence of FCGS may vary in different regions. Food residues predispose to microbial proliferation and plaque accumulation which in turn triggers oral inflammation. Affected cats experience oral pain that causes salivation, halitosis and inability to groom.

In this survey, the 3–4 age group seems to be most commonly presented, accounting for 32.79% of the study population. One study showed that the median age of cats with FCGS was 8 years and 5 months, and the age distribution ranged from 1 to 20 years (28). This may be due to the difference in the age distribution of cats treated in hospitals. The results of this study showed that there was no significant correlation between FCGS and sex. The breeds of cats with FCGS were evenly represented in the whole sample population. The number of Chinese pastoral cats raised in Xi’an was relatively high, which was also reflected in the fact that Chinese pastoral cats accounted for the greatest proportion of affected cats.

In this survey, there were significant differences between the unimmunized, not fully immunized and immunized cats between cats with and without FCGS (*p* < 0.01), suggesting that there was a problem of incomplete immunization of the infected cats, which may be related to the fact that 42.62% of the cases in this sample had a history of homelessness, resulting in fewer cases receiving vaccinations according to the vaccination program. According to Zhou et al. (29) current commercial vaccines do not appear to provide enough cross protection. Maybe, inadequate immunization status has only a secondary role in the percentage of affected stray cats (29). Studies have shown that the incidence of FCGS is significantly greater in multicat households than in single-cat households, and the incidence of FCGS increases with the number of cats in the household (30). Among the 61 cats with FCGS investigated in this study, the number of cases from multicat households was relatively high.

Despite its common occurrence, the aetiology of chronic gingivostomatitis in cats remains uncertain. Aetiology is likely multifactorial, and several infectious agents may be associated with chronic gingivostomatitis (5, 31, 32). In this study, the FCV detection rate of cats with FCGS was the highest, which was much greater than the normal FCV carrier rate, suggesting that there may be a certain correlation between FCV infection and FCGS. However, a study of 26 cats with FCGS revealed FIV positivity in 15% and FeLV positivity in 34.6%, with no FCV detected in oral swab samples from these 26 cats (33). Different studies in different regions have shown great differences in results; thus, it is speculated that the occurrence of FCGS is not caused by a single factor and that infectious factors, autoimmune factors, the living environment, stress and other factors may play their respective roles in the pathogenesis of FCGS. Clinicians should fully consider various pathogenic factors and adopt comprehensive means to carry out effective treatment when treating cats with FCGS to achieve more satisfactory effects.

Dental X-ray results revealed that alveolar bone loss was severe and extensive in cats with FCGS. Residual roots after tooth extraction are one of the important causes of FCGS recurrence (34). Therefore, obtaining dental x-rays before dental procedures in order to detect possible tooth resorption, and after extractions to confirm complete root removal, are effective measures to improve cure rate.

Histologically, the oral mucosa of healthy cats was composed of squamous epithelium with few inflammatory cells, while specimens obtained from FCHS cats revealed significant inflammatory infiltration, mainly composed of plasma cells and lymphocytes. Consistent with the results of this study, it has been reported that the inflammatory cells infiltrating the oral mucosa of cats with FCGS are mainly plasma cells and lymphocytes, and sometimes neutrophils and eosinophils are also present (35), suggesting that there may be viral infection in cats with FCGS.

### Oral microbiota analysis

A total of 16 cats with FCGS and 6 healthy controls were included in this study. 16S rDNA amplicon sequencing technology was used to study the difference in oral microbial composition between cats with FCGS and healthy cats, as well as the difference in oral microbial composition before and after tooth extraction. In this study, the overall number of ASVs in cats with FCGS was greater than that in healthy cats. The number of ASVs in cats with FCGS decreased after tooth extraction, indicating that cats with FCGS have more diverse oral microbes than healthy cats. Alpha diversity analysis revealed that the oral microbial community diversity of cats with FCGS was greater than that of healthy cats, and the oral microbial community diversity of cats with FCGS differed before and after tooth extraction. The results of the beta diversity analysis showed that there were significant differences in the beta diversity of the oral microbial communities between cats with FCGS and healthy cats; that is, the diversity distance between oral microbial flora samples was large, and the degree of differentiation between microbial communities was significantly different. This indicates that the oral microbial community structure of cats changes significantly during the progression from oral health to chronic gingivitis. Second, the two clusters of cats with FCGS before and after tooth extraction partially overlapped. This indicates that the oral microbial community structure of cats with FCGS only partially changed following tooth extraction.

At the phylum level, the three most common oral microorganisms in cats with FCGS were *Firmicutes*, *Bacteroidota,* and *Proteobacteria*. At the genus level, the most abundant bacteria were *Porphyromonas*, followed by *Treponema*. It has been reported that the Gram-negative anaerobic bacteria *Actinomyces*, *Bacteroides intermedia* and *Bacteroides gingivoides* have been detected in cats with FCGS through serological reactions (36). The latter two belong to *Bacteroidota*, which is consistent with the results of this study. Nakanishi et al. (28) reported that anaerobic bacteria may be the main factor affecting the oral environment of cats with FCGS. In this study, the abundance of anaerobic bacteria in the mouths of the cats decreased but was still greater than that of the healthy cats 1 month after tooth extraction. This indicates that the abundance of anaerobic bacteria in the mouth can be effectively reduced, and the oral environment can be improved by tooth extraction.

The functional prediction results showed that at the third level, the two-component signaling system was enriched in cats with FCGS, which enabled the bacteria to sense and respond to environmental stimuli and changes and thus adapt to the living environment (37). At the same time, enriched quorum sensing in cats with FCGS regulates virulence, as it participates in controlling the production of bacterial virulence factors and biofilms, which can lead to host inflammation (38). In addition, a variety of genes related to amino acid metabolism and glucose metabolism were enriched in the cats with FCGS, suggesting their potential importance in the pathogenesis of FCGS.

### Pathogen analysis of FCGS

LEfSe analysis revealed that the relative abundances of *Treponema* and *Fusobacterium* in cats with FCGS were significantly greater than those in healthy cats. Moreover, the abundances of *Porphyromonas*, *Treponema* and *Fusobacterium* in cats with FCGS decreased significantly after tooth extraction. Therefore, *Porphyromonas*, *Treponema* and *Fusobacterium* have been speculated to play important roles in the development of FCGS, and all three are anaerobic bacteria. One study also revealed that the most abundant bacterial genera in the FCGS mouth included *Treponema*, *Bacteroides,* and *Porphyromonas* (39).

*Porphyromonas*, a genus of Gram-negative oral obligate anaerobic bacteria, was most abundant in cats with FCGS. It plays a key role in the pathogenesis of periodontitis, can also express various virulence factors, can destroy periodontal tissue, and is associated with systemic diseases (40, 41). Its virulence factors affect host immune cell function and can induce autophagy in different types of cells, including endothelial cells, gingival fibroblasts, macrophages, and dendritic cells (42). In addition, *Porphyromonas* species make oral inflammation difficult to control, further damage the epithelial barrier, and achieve intracellular self-reproduction within epithelial cells, which also allows them to evade the host immune system (43). *Porphyromonas* may play a key role in the development of FCGS disease. In this study, a clinically isolated strain homologous to *Porphyromonas gulae* was obtained by the isolation and culture of oral samples from infected cats. This strain exhibited multienzyme activity. These biochemical metabolic reactions maintain not only the survival and proliferation of bacteria but also the activity of indolase. These activities may also mediate interactions between bacteria and between bacteria and their host and may even cause cytotoxicity to host tissues.

### Analysis of drug sensitivity

The treatment of chronic gingivitis usually involves the use of antibiotics. After tooth extraction surgery, it is also important to choose appropriate antibiotics and follow a standardized medication procedure to avoid the development of drug resistance. Drug sensitivity testing on the oral microbiota of cats with FCGS showed that enrofloxacin had a good antibacterial effect on both aerobic and anaerobic bacteria *in vitro*. The oral microbiota may already be resistant to metronidazole. The results of oral microbiota drug sensitivity vary greatly among individuals and are affected by individual drug use history. However, summarizing the general sensitivity trends can provide a reference for clinical drug use. Combined with the previous analysis, the proportion of anaerobic bacteria in the oral microbiota of cats with FCGS increased; therefore, it is recommended to select antibiotics that are sensitive to anaerobic bacteria in the antibacterial spectrum.

In this study, the MIC of the isolated *Porphyromonas* strain was determined. The strain was sensitive to enrofloxacin, moderately sensitive to gentamicin, amoxicillin and ceftriaxone sodium, and resistant to cephalexin, doxycycline hydrochloride and metronidazole. Enrofloxacin is recommended as the first choice for FCGS.

## Conclusion

THE incidence of FCGS in cats in Xi’an was not significantly correlated with age, sex or breed. However, it was associated with immunization, a history of homelessness and multicat rearing environments. The number of neutrophils and the serum amyloid A concentration were increased, and the percentage of cats with FCGS positive for calicivirus antigen was high. All cats had different degrees of dental calculus, alveolar bone level loss or tooth resorption problems. The abundance of potential pathogens in the mouths of cats with FCGS was increased, and the abundance of anaerobic bacteria was significantly increased. *Porphyromonas*, *Treponema* and *Fusobacterium*, which may be potential pathogens of FCGS, were enriched in the mouths of cats with FCGS. The composition of the oral microbiota in cats tended to be normal after tooth extraction. An isolated strain was obtained from the mouths of the infected cats and was homologous to *Porphyromonas gulae*. Both the FCGS-associated oral microbiota and the isolated strain were highly sensitive to enrofloxacin.

## Data availability statement

The datasets generated and/or analyzed during the current study are available in the NCBI Short Read Archive (SRA) with PRJNA1092748.

## Ethics statement

The animal studies were approved by Animal Ethical and Welfare Committee of Northwest Agriculture and Forest University. The studies were conducted in accordance with the local legislation and institutional requirements. Written informed consent was obtained from the owners for the participation of their animals in this study.

## Author contributions

PD: Writing – review & editing, Writing – original draft, Methodology, Data curation. MY: Writing – original draft, Methodology, Data curation. JD: Writing – original draft, Validation, Data curation. KW: Writing – original draft, Validation, Data curation. RC: Writing – original draft, Validation, Data curation. XF: Writing – original draft, Validation. CC: Writing – original draft, Validation. XZ: Writing – review & editing, Project administration, Methodology, Funding acquisition.
